# Endovascular Therapy vs. Thrombolysis in Pre-stroke Dependent Patients With Large Vessel Occlusions Within the Anterior Circulation

**DOI:** 10.3389/fneur.2021.666596

**Published:** 2021-06-02

**Authors:** Andreas Kastrup, Christian Roth, Maria Politi, Maria Alexandrou, Helmut Hildebrandt, Andreas Schröter, Panagiotis Papanagiotou

**Affiliations:** ^1^Department of Neurology, Klinikum Bremen-Mitte, Bremen, Germany; ^2^Department of Neuroradiology, Klinikum Bremen-Mitte, Bremen, Germany; ^3^Department of Radiology, Aretaieion Hospital, School of Medicine, National & Kapodistrian University of Athens, Athens, Greece

**Keywords:** stroke, thrombolysis, thrombectomy, outcome, endovascular, pre-stroke disability

## Abstract

**Background:** In the past few years, several randomized trials have clearly shown that endovascular treatment (ET) in addition to intravenous thrombolysis (IVT) is superior to IVT alone in patients with proximal cerebral arterial occlusions. However, the effectiveness of ET in pre-stroke dependent patients (modified Rankin Scale ≥3) is uncertain.

**Methods:** Using our prospectively obtained stroke database, we analyzed the impact of pre-stroke dependence on the rates of poor outcome (discharge mRS 5–6), in-hospital death, infarct sizes, and symptomatic intracranial hemorrhage (SICH) in patients with distal intracranial carotid artery M1 and M2 occlusions during two time periods.

**Results:** From 1/2008 to 10/2012, a total of 544 patients (455 without and 89 with dependence) were treated with IVT, and from 11/2012 to 12/2019 a total of 1,061 patients (919 without and 142 with dependence) received ET (with or without IVT). Irrespective of the treatment modality, the dependent patients had significantly higher rates of poor outcome (55 vs. 32%, *p* < 0.001), death (24 vs. 11%; *p* < 0.001), or SICH (8.2 vs. 3.6%, *p* < 0.01) than independent patients. In dependent patients, ET significantly reduced the rates of poor outcome (49 vs. 64%, *p* < 0.01) and led to smaller infarcts, whereas the rates of in-hospital death (25 vs. 22%; *p* = 0.6) or SICH (8.5 vs. 7.9%, *p* = 0.9) were comparable between both treatment modalities.

**Conclusions:** Compared with IVT, ET avoids poor outcome and leads to smaller infarcts in dependent patients. However, the overall high rates of poor outcome in this patient population stress the importance to perform decisions based on a case-by-case basis.

## Introduction

With the publication of five landmark trials in the year 2015, endovascular treatment (ET), in addition to intravenous thrombolysis (IVT), has become an established treatment for acute stroke patients with proximal large vessel occlusions (1–5). In these trials, the benefit of ET in addition to IVT on outcome was principally observed across all patient groups studied. However, patients with pre-existing dependency were not included in the aforementioned large, controlled, randomized ET trials. Therefore, current stroke guidelines only state that ET may be reasonable in this patient population (6). Since pre-stroke disability will continue to rise in the coming years (7), the potential impact of ET on outcome in patients with prior disabilities has received increased attention. In fact, several recent case series as well as an analysis of the MR Clean Registry have revealed favorable functional and procedural outcome rates after ET in patients with prior disabilities compared to those without pre-stroke disability (8–11), yet all of these studies lacked a control group, such that the true treatment effect of ET in pre-stroke disabled patients is unknown.

Therefore, we analyzed the impact of preexisting dependency on clinical outcome at the time of discharge as well as the radiological outcome in patients with anterior circulation large vessel occlusions during two time periods using our prospectively obtained database of a high-volume stroke center in which patients with prior disabilities [except for severely affected patients, i.e., bedridden patients with a modified Rankin Score (mRS) of 5] are not routinely excluded from ET or IVT. During the first time period, all patients had been treated with systemic thrombolysis, whereas an endovascular therapy with stent retrievers (with or without systemic thrombolysis) was routinely used during the second time period.

## Methods

### Study Population

The study population comprised all acute stroke patients with a proximal large vessel occlusion within the anterior circulation (distal intracranial carotid artery and/or M1 and/or M2 segments of the middle cerebral artery) and who had been treated from January 2008 through December 2019. The main exclusion criteria were (1) distal occlusions beyond the M2 segment, (2) posterior circulation strokes, and (3) bilateral strokes. From January 2008 to November 2012, all patients were treated with systemic thrombolysis alone, mainly using the inclusion and exclusion criteria, as well as a drug dose for thrombolytic treatment of the NINDS study protocol. At our institution, patients can be treated up to 4.5 h after symptom onset, and there is no upper age limit for eligibility.

Endovascular treatment with or without systemic thrombolysis was performed after November 2012 in all patients who presented within 6 h (within 4.5 h in patients additionally treated with rt-PA) of symptom onset (using no upper age limit or specific imaging exclusion criteria).

In each patient, the following demographic data and stroke risk factors were collected: age, gender, arterial hypertension, diabetes, and atrial fibrillation. The time to thrombolysis (or to thrombectomy in patients without prior systemic thrombolysis) from stroke onset was also noted.

Information on the pre-stroke living situation was obtained from the patients themselves, their families, their relatives, or their primary care physicians as well as outpatient medical personnel. Patients who were dependent on the daily help of others before the stroke (either coming from home or coming from a nursing home) were classified as dependent, reflecting a mRS of 3–4; all other patients were classified as independent (mRS 0–2). At our institution, bedridden patients with severe disabilities, i.e., with a mRS of 5, are usually not treated with ET. Since the exact pre-stroke mRS is often misjudged, especially in the emergency setting, dependent patients were not further subcategorized between a mRS value of 3 or 4 (12, 13).

The protocol of our stroke registry had been approved by our local ethics committee. Because of the retrospective character of this study, the lack of treatment influence, and the clinical data having been collected as part of a national quality control program, the study was exempted from informed consent. Using our stroke database, the clinical outcomes of patients treated before December 2014 as well as a subgroup analyses have been published previously (14–17).

### Imaging Techniques

Non-enhanced CT (NCT) and CT angiographic acquisitions before treatment were performed on a 4-row Multisection CT scanner (Siemens Volume Zoom, Siemens Medical Solutions, Forchheim/Germany).

NCT was performed with the patient in a head holder in the transverse plane. Using the following parameters, incremental CT acquisitions of the brain were obtained: 120 kVp, 250 mA, 2-s scan time, and 5-mm section thickness.

To allow visualization of the vascular tree from the distal common carotid artery to the intracranial vessels, the CT angiography covered the region from the fifth vertebral body up to the vertex. The following parameters were used: 120 kVp, 200 mAs, 4x 1-mm collimation, 5.5 mm/rotation table feed, and 0.5 s rotation time. A total of 100 ml of contrast material was injected intravenously at a flow rate of 4 ml/s using a power injector. For follow-up studies, repeated CT or MR scans were obtained after 1–3 days after treatment or immediately in case of clinical worsening.

### Image Analysis

CT and MR image analyses were performed jointly by a board-certified neuroradiologist (PP, 20 years of neuroimaging review experience) and a stroke neurologist (AK, with 18 years of neuroimaging review experience) on a high-resolution monitor.

Since CTA source images are superior to non-contrast CT images to detect early ischemic changes, they were used to determine the Alberta Stroke Programme Early CT score (CTA-SI-ASPECTS) (16). The follow-up CT or MR scans were used to determine the final ASPECTS as a marker of infarct extent as well as the incidence of symptomatic intracranial hemorrhages (SICH) using the ECASS III definition.

### End Points and Analyses

Functional outcome was assessed by a senior vascular neurologist who was certified for NIHSS and mRS scoring (AK, AS). The mRS at the time of hospital discharge was used for early clinical outcome analyses. The main outcome measures were a poor clinical outcome (discharge mRS of 5–6), in-hospital death, infarct sizes, and SICH rates.

In a first step, we compared the clinical and radiological outcomes between pre-stroke dependent (mRS ≥3) and independent (mRS 0–2) patients after either IVT or ET. In a second step, we then analyzed the impact of ET on the clinical and radiological outcomes in pre-stroke dependent patients compared to IVT.

### Statistical Analysis

Continuous values were expressed as mean ± standard deviation or as median ± interquartile range (IQR). Nominal variables were expressed as count and percentages. For comparisons of categorical data, two-tailed chi-square statistics with Yates correction and univariate Fisher's exact test were used. Fisher's exact test was used when the predicted contingency table cell values were less than five. Analyses of continuous variables were performed with an unpaired Student's *t*-test or, in case of abnormally distributed data, with a Mann–Whitney *U*-test.

A stepwise, forward, multiple-regression analysis (*P* in 0.05, *P* out 0.1) was applied to determine the independent predictors of a poor clinical outcome (mRS 5–6). The following variables were considered: age, sex, treatment modality, baseline NIHSS, baseline and follow-up SI-ASPECTS, diabetes, hypertension, atrial fibrillation, prior stroke, occlusion site, and SICH. Results are presented as odds ratios (ORs) with 95% confidence interval.

A value of *p* < 0.05 was considered to indicate a statistically significant difference. All statistical analyses were performed with SPSS (version 22, SPSS Inc.).

The data that support the findings of this study are available from the corresponding author upon reasonable request.

## Results

### Demographic Data

The IVT group comprised a total of 544 patients, and 1,061 patients had received ET, respectively. Among the IVT patients, 455 (84%) were previously independent and 89 (16%) were dependent before stroke. In the ET group, 919 (87%) patients were functionally independent before the current stroke, whereas 142 (13%) patients were dependent of the help of others before their stroke. Table 1 shows a summary of the baseline characteristics of the entire study population.

**Table 1:** Baseline characteristics.

**Number of patients**	***n* = 1,605**
Age	
Mean (years)	74 ± 12
Hypertension	1,306 (81)
Atrial fibrillation	879 (55)
Diabetes	335 (21)
NIHSS	
Median, IQR	14 (10–18)
Pre-stroke mRS (0–2)	1,374 (86)
Pre-stroke mRS (≥3)	231 (14)
Imaging	
Location of occlusion^a^	
ICA	298 (19)
M1 MCA	948 (59)
M2 MCA	359 (22)
Baseline SI-ASPECTS	
Median, IQR	8 (7–10)
Treatment	
Alteplase alone, iv	544
Endovascular therapy (with or without thrombolysis)	1,061

a *Most proximal occlusion location*.

*NIHSS, National Institutes of Health Stroke Scale; SI-ASPECTS, Source Image Alberta Stroke Program Early CT Score (only for anterior circulation strokes); ICA, internal carotid artery; MCA, middle cerebral artery*.

### Overall Clinical and Radiological Outcomes

After either IVT or ET, a total of 568/1,605 patients (35%) had a poor outcome (mRS 5–6), and 212/1,605 (13%) died in the hospital. Overall, 68 patients (4.2%) developed a SICH using the ECASS III definition. The rates of poor outcome (55 vs. 32%, *p* < 0.001), in-hospital death (24 vs. 11%; *p* < 0.001), and SICH (8.2 vs. 3.6%, *p* < 0.01) were significantly higher in previously dependent patients than in independent patients. The infarct sizes were comparable between both treatment groups (median 7; IQR: 4–8 vs. median 7; IQR 4–8; *p* = 0.5).

### Pre-stroke Dependent Patients

Table 2 shows a summary of the baseline characteristics of pre-stroke dependent patients after either IVT or ET. Notably, the patients treated with ET were significantly younger but more severely affected than those treated with IVT, respectively.

**Table 2:** Clinical characteristics of previously dependent stroke patients (mRS ≥3) after either thrombolysis or endovascular therapy.

	**Intravenous thrombolysis**	**Endovascular treatment**	***p*-value**
	***n* = 89**	***n* = 142**	
Age			
Mean (years)	86 ± 7	83 ± 8	*p* < 0.05
Hypertension	81 (91)	130 (91)	
Atrial fibrillation	54 (61)	79 (56)	
Diabetes	21 (24)	43 (30)	
Prior stroke	22 (25)	52 (37)	*p* = 0.06
NIHSS (median, IQR)	15 (11–18)	17 (13–20)	*p* < 0.05
Location of occlusion			
ICA ± M1 MCA	64 (72)	105 (74)	
M2 MCA	25 (28)	37 (26)	
Baseline SI-ASPECTS (median, IQR)	8 (6–10)	8 (6–10)	

*NIHSS, National Institutes of Health Stroke Scale; SI-ASPECTS, Source Image Alberta Stroke Program Early CT Score (only for anterior circulation strokes); ICA, internal carotid artery; MCA, middle cerebral artery*.

The clinical and imaging outcome data are given in Table 3. Compared with IVT, endovascular treatment significantly reduced the rates of poor outcome (49 vs. 64%; OR_unadjusted_, 0.5; 95% confidence interval, 0.32–0.94; *p* < 0.05). The rates of in-hospital death did not differ significantly between and ET and IVT (22 vs. 25%, *p* = 0.6).

**Table 3:** Clinical and imaging outcomes after intravenous thrombolysis (IVT) or endovascular treatment (ET) in previously dependent patients at the time of discharge.

	**IVT**	**ET**	***p*-value**
	***n* = 89**	***n* = 142**	
mRS 5–6	57 (64)	70 (49)	<0.05
Death	22 (25)	31 (22)	0.6
Infarct size^a^ (median, IQR)	5 (2–8)	7 (5–9)	<0.01
SICH	7 (8)	12 (8)	0.9

*mRS, modified Rankin scale; SICH, symptomatic intracerebral hemorrhage*.

a *Using the follow-up Alberta Stroke Program Early CT Score*.

The infarct sizes were significantly smaller after ET than after IVT (median: 7; IQR: 5–9 vs. median: 5; IQR: 2–8; *p* < 0.01), whereas the SICH rates were comparable between both treatment groups (8.5 vs. 7.9, *p* = 0.9).

In the multivariate regression analysis, admission NIHSS and endovascular treatment were significantly associated with a poor clinical outcome (Table 4).

**Table 4:** Multivariable odds ratios and 95% confidence intervals of poor outcome in dependent patients.

	**Odds ratio**	**CI lower**	**CI higher**	***p*-value**
Age^a^	1.02	0.99	1.06	0.3
Atrial fibrillation	0.8	0.49	1.31	0.4
Diabetes	1.53	0.81	2.87	0.2
Prior stroke	1.09	0.57	1.98	0.77
Initial NIHSS^b^	1.12	1.05	1.18	<0.001
ET	0.43	0.24	0.76	<0.01

*CI, confidence interval; NIHSS, National Institutes of Health Stroke Scale; ET, endovascular therapy*.

a *Per 1 year increase*.

b *Per one point increase*.

## Discussion

The main purpose of this study was to analyze the impact of endovascular therapy on clinical and radiological outcomes in patients with pre-stroke disability and proximal large vessel occlusions within the anterior circulation compared with systemic thrombolysis. After either treatment modality, the dependent patients had worse clinical and radiological outcomes than the independent patients. Compared with IVT, endovascular therapy improves the early clinical outcome, avoids poor outcome, and leads to smaller infarcts in patients with prior disabilities.

In our study cohort, 16% of all patients were dependent on the daily help of others before stroke, supporting the notion that these patients represent a significant proportion of the acute ischemic stroke population. Furthermore, 11% of the patients within the MR CLEAN registry were likewise pre-stroke dependent (9). Comparable rates were also found in single-center studies from Israel or from Sweden (11, 18). Studies focusing on patients treated with thrombolysis alone and with unknown vessel status reported proportions of patients with pre-stroke disability ranging from 10 to 30% (19, 20).

Despite the relatively high numbers of patients with acute large vessel occlusions and pre-stroke disabilities in everyday clinical practice, data from randomized trials dealing with the impact of ET compared with IVT in this patient population is scarce and also likely reflects a selection bias. In the Solitaire With the Intention for Thrombectomy as Primary Endovascular Treatment for Acute Ischemic Stroke trial, only three patients (1.5%) were functionally dependent before their current stroke, while in the Endovascular Recanalization With Solitaire Device vs. Best Medical Therapy in Anterior Circulation Stroke Within 8 h trial, there was only one patient (0.5%), respectively (2, 3).

Recently, several single-center studies and registries have reported the beneficial effects of ET in pre-stroke dependent patients (8, 9, 11, 18). In fact, all of these studies were able to show that pre-stroke disability does not significantly reduce the likelihood to return to the pre-stroke functional level after ET. However, none of these studies had incorporated a control group. We therefore extend these findings and show that ET increases the odds to avoid a poor outcome in this patient population compared with IVT. Taken together, these data support the notion that previously dependent patients should not be routinely excluded from ET.

Despite the overall positive effect of ET on clinical outcome, pre-stroke dependent patients had significantly higher rates of in-hospital death than independent patients irrespective of the treatment modality in our case series. In good agreement with this finding is the fact that the mortality rates at 90 days were significantly higher in patients with a pre-stroke mRS of 2–3 (40%) than independent patients (14%) after endovascular therapy in a recent analysis of two large comprehensive stroke centers in the US (8). In the MR Clean Registry, the mortality rates were twice as high in dependent than in independent patients at 3 months after ET (9). These results emphasize that pre-stroke dependent patients have a higher risk of suffering death after ET, likely reflecting higher rates of comorbidities in this patient population. Although not the focus of our study, but in support of this notion, the pre-stroke dependent patients had significantly more comorbidities than independent patients in the aforementioned MR Clean Registry and analysis of the two comprehensive stroke centers (8, 9). Aside from a higher disease burden caused by preexisting comorbidities, the dependent patients are more likely to have other complications than SICH. In addition, withdrawal of care according to their preferences could also come into play more often.

The overall rates of SICH were 4.1% after ET or 4.4% after IVT, which is within the magnitude of SICH rates observed in the large, randomized trials (2, 4). In contrast to these studies, however, the SICH rates were significantly higher in dependent patients compared to independent patients in our cohort. Higher rates of SICH have also been likewise reported in the MR Clean Registry in a comparable patient population (9).

Notably, ET did not affect the incidence of SICH in patients with prior disabilities, stressing its safety in this patient population.

In this study, we also analyzed the impact of ET on infarct sizes in dependent patients compared with IVT. Using the follow-up ASPECTS as a marker of infarct extent, we observed significantly smaller infarct sizes after ET than after IVT in dependent patients. These data corroborate previous findings, albeit mainly in independent patients, in which the final infarct volumes were significantly smaller after ET than after IVT (21, 22). Although it has clearly been shown that a reduced final infarct volume only partially mediates the positive effect of ET on clinical outcome (21), our primary finding of an improved clinical outcome after ET in dependent patients is thus also supported by the imaging data.

This study has certain limitations. First, the data were obtained retrospectively in a single academic center and in a non-randomized fashion. This limits the generalizability of our results, which need to be corroborated in a randomized controlled trial. Second, the causes for preexisting disability was not obtained in our study, all the more considering that dependent patients likely harbor complex comorbid conditions. Third, we used the mRS at the time of discharge to determine the clinical outcome instead of the widely accepted 90 days. In support of this approach, a reanalysis of the NINDS tPA stroke trial revealed a strong correlation between the day 7 mRS and the 90-day mRS (23). Finally, the impact of recanalization on clinical and radiological outcomes could not be incorporated into our analyses since information on these important variables was not available in the IVT group.

## Conclusions

In everyday clinical practice, a substantial proportion of acute stroke patients with large vessel intracranial vessel occlusions have preexisting disabilities. In these patients, ET can be performed safely and avoids a poor outcome compared with IVT. However, the high death rates in this patient population stresses the importance to perform decisions based on a case-by-case basis.

## Data Availability Statement

The raw data supporting the conclusions of this article will be made available by the authors, without undue reservation.

## Ethics Statement

The studies involving human participants were reviewed and approved by Local Ethics Committee of the Ärztekammer Bremen. Written informed consent for participation was not required for this study in accordance with the national legislation and the institutional requirements.

## Author Contributions

AK, CR, and PP contributed to the study design, data acquisition, critical data review and interpretation of data, and contributed to the primary manuscript writing and tables. CR, MP, MA, HH, and AS contributed to data acquisition and interpretation of data. AK, CR, MP, MA, HH, AS, and PP contributed to the critical revision and final approval of the manuscript. All authors contributed to the article and approved the submitted version.

## Conflict of Interest

The authors declare that the research was conducted in the absence of any commercial or financial relationships that could be construed as a potential conflict of interest.
